# High Suitcordance for Panvascular Full-Watershed Organs: A New Interventional Perspective

**DOI:** 10.34133/research.0974

**Published:** 2025-10-29

**Authors:** Lingsen You, Yuheng Chen, Zeyang Zhang, Yu Wang, Li Shen, Junbo Ge

**Affiliations:** ^1^ Department of Cardiology, Shanghai Institute of Cardiovascular Diseases, Zhongshan Hospital, Fudan University, Shanghai 200032, China.; ^2^ National Clinical Research Center for Interventional Medicine, Shanghai 200032, China.; ^3^ Oriental Pan-Vascular Devices Innovation College, University of Shanghai for Science and Technology (USST), Shanghai 200093, China.; ^4^ State Key Laboratory of Cardiovascular Diseases, Zhongshan Hospital, Fudan University, Shanghai 200032, China.; ^5^ NHC Key Laboratory of Ischemic Heart Diseases, Shanghai 200032, China.; ^6^ Department of Cardiology, Shidong Hospital, Shanghai 200438, China.

## Abstract

Panvascular medicine underscores the integration of vascular networks across organs such as the heart, brain, kidneys, and limbs into a unified system. In this system, metabolic aberrations, endothelial dysfunction, and hemodynamic disturbances in one organ can drive synergistic pathologies elsewhere. However, current interventional device development has largely overlooked richly vascularized, high-perfusion organs like the liver and kidneys. Furthermore, the pervasive challenge of “low suitcordance”—a term we introduce to describe suboptimal device performance over its entire life cycle—confronting interventional devices for panvascular full-watershed organs remains unresolved. (Here, the term “full-watershed” metaphorically denotes organs that, like geographical watersheds, receive marked perfusion from the systemic circulation, emphasizing their collective role in panvascular health.) This article introduces “suitcordance” (short-term suitability and long-term concordance) as a novel framework for evaluating device performance that transcends traditional metrics like biocompatibility. We propose that interdisciplinary innovation, fusing materials science, biomechanics, mechanobiology, and artificial intelligence can address this gap. The Xinsorb bioresorbable scaffold illustrates a path toward “high suitcordance” devices, offering a paradigmatic reference for interventions in cerebral, peripheral, hepatic, and renal vasculatures. This approach provides a new paradigm for advancing interventional devices from isolated vascular repair to the synergistic management of multivascular bed lesions and the restoration of systemic functional equilibria.

## “Low Suitcordance” Dilemma: A Systemic Barrier in Panvascular Intervention

In medicine, traditional disciplinary boundaries are blurring. In 2022, the *European Heart Journal* published a special review on panvascular medicine, sparking global scholarly interest in the systemic management of vascular diseases. The definition of panvascular diseases was thereby clarified: systemic vascular disorders characterized by atherosclerosis as a common pathological hallmark, predominantly affecting critical organs such as the heart, brain, kidneys, and limbs [1]. The theoretical framework of Panvascular Medicine has since solidified, signifying a paradigmatic shift in our understanding of vascular diseases from organ-specific fragmentation to systemic integration.

For the comprehensive treatment of panvascular diseases, interventional therapies have emerged as a cornerstone of management, owing to their minimal invasiveness, rapid recovery, and cost-effectiveness [2]. From percutaneous coronary intervention using drug-eluting stents (DES) to stent-assisted coil embolization for cerebral aneurysms, innovative interventional devices are steadily propelling systemic treatments across coronary, cerebral, and peripheral vasculatures.

Nevertheless, on the path toward revolutionary advancements for panvascular full-watershed organs (encompassing the vasculatures of the heart, brain, limbs, liver, kidneys, lungs, and spleen; while these organs exhibit distinct vascular architectures, their shared dependence on high-volume perfusion and susceptibility to systemic hemodynamic perturbations justifies their collective consideration under the panvascular umbrella, though future implementations may benefit from organ-specific subclassifications), a pressing challenge persists: the “low suitcordance” dilemma.

## The Concept of Suitcordance: Beyond Biocompatibility

To address this challenge, we must first establish a more comprehensive evaluation framework. While terms like “biocompatibility” or “mechanical matching” are useful, they fail to capture the full spatiotemporal dynamics of a device’s life cycle. Therefore, we propose the concept of suitcordance, defined as short-term suitability and long-term concordance, to quantify interventional device performance and guide future development [3]. In clinical practice, we have preliminarily delineated key evaluation metrics within a conceptual formula:

Γsc=α·ΨSTS+β·ΦLTC (1)

While currently a conceptual guide, this framework is designed for future operationalization through machine learning models. Such models could integrate high-dimensional clinical, imaging, and biomechanical data to quantify these parameters and derive patient-specific predictions. Here, Γsc is a score where a higher value signifies better overall device performance. ΨSTS (short-term suitability) assesses immediate device–host interactions, including device–biological interface compatibility and dynamic mechanical property matching, while ΦLTC (long-term concordance) evaluates the synchrony of material degradation with benign vascular remodeling and the minimization of systemic perturbations.

ΨSTS would be derived from immediate postprocedural metrics—such as thrombogenicity potential, device deployment accuracy, and hemodynamic compatibility—extracted from procedural imaging, angiography, and intravascular assessments. ΦLTC, conversely, would be quantified from serial longitudinal data, including structural changes (e.g., lumen patency and tissue coverage), functional recovery (e.g., vasomotion), and systemic biological responses (e.g., inflammatory or metabolic profiles). This data-driven approach aims to make Γsc a patient- and lesion-specific predictor of device success.

For example, when evaluating the suitcordance (Γsc) of the renal artery stent, the following points need to be considered: acute thrombogenicity rate; immediate strut apposition rate; conveyor system possibility (ΨSTS) and late lumen loss; recovery of endothelial vasomotion; and changes in inflammatory markers related to degradation by-products (ΦLTC).

The organ-disease-specific weighting coefficients, α and β, highlight that the relative importance of short-term versus long-term performance varies.

Crucially, the weighting coefficients α and β are not fixed but can be empirically derived from real-world clinical data. For instance, machine learning models could analyze large-scale registries to determine that for patients with acute, life-threatening lesions (e.g., ST-segment elevation myocardial infarction), α should be heavily weighted, whereas for stable, progressive diseases (e.g., chronic limb-threatening ischemia), β would carry greater importance. This data-driven calibration ensures that the Γsc score is dynamically tailored to specific clinical scenarios, patient risk profiles, and treatment priorities, bridging the conceptual framework to practical application. For instance, in acute ischemic events like hepatic artery embolism, the short-term suitability coefficient, α, is paramount. Conversely, in treating chronic, stable lesions, the long-term concordance coefficient, β, dominates.

Achieving high ΨSTS implies that a device, upon implantation, perfectly matches local vascular anatomy and hemodynamics. Achieving high ΦLTC means the device guides benign remodeling over time, ensuring the long-term restoration of 3 key ecological equilibria within the panvascular system: cellular (e.g., promoting endothelial repair over smooth muscle cell proliferation, etc.), mechanical (e.g., restoring natural vasomotion and avoiding stress concentration, etc.), and physicochemical-immune (e.g., minimizing chronic inflammation from device materials or by-products, etc.) [4]. Essentially, suitcordance is the device’s manifest performance, governed by its ability to restore and maintain homeostasis. Predictive modeling using artificial intelligence (AI), particularly through patient-specific “digital twins”, will be instrumental in simulating these equilibria in silico to optimize device designs preclinically.

**Fig. 1.**
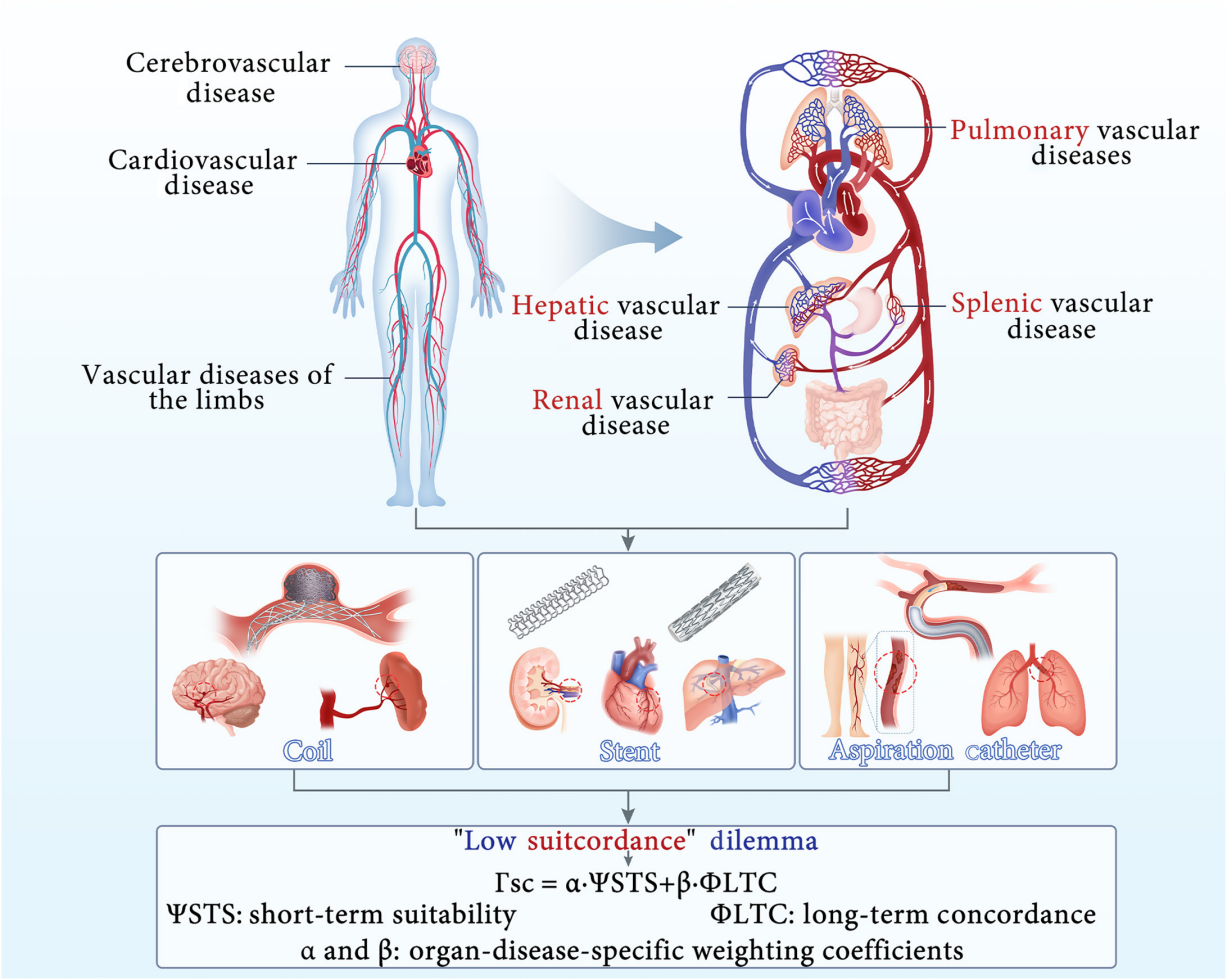
The “low suitcordance” dilemma in panvascular full-watershed organs. While innovation has centered on cardiac, cerebral, and peripheral vasculatures, organs like the liver and kidney represent a critical frontier for developing “high suitcordance” devices that achieve both short-term suitability and long-term concordance.

Concurrently, we contend that prior research has disproportionately focused on cardiac, cerebral, and limb vasculatures [5], leading to insufficient emphasis on interventional devices for other panvascular full-watershed organs (liver, kidneys, lungs, and spleen). For diseases affecting these organs, devices must be developed under a panvascular medicine lens to facilitate multidisciplinary innovation (Fig. 1).

## The Liver and Kidneys: Current Limitations and Challenges in Interventional Therapy

Taking the liver and kidneys as examples, their unique vascular architectures and hemodynamic profiles play distinct roles in maintaining the 3 systemic equilibria mentioned earlier [6–10]. These organs also share endothelial injury mechanisms (e.g., mitochondrial dysfunction driving oxidative stress [11]) with the broader systemic vasculature.

However, current interventional modalities for these organs—such as transjugular intrahepatic portosystemic shunt (TIPS) for portal hypertension or coil embolization for renal arteriovenous malformations—face substantial challenges. They often not only struggle to achieve short-term procedural success (ΨSTS) but also fail to secure long-term balance in the human body (ΦLTC), thereby compromising systemic vascular function.

Consider vascular stenting for hepatic artery stenosis. The hepatic artery’s small diameter and tortuous path mean that while DES outperform bare-metal stents, the risks of restenosis and thrombosis remain high [12]. In parallel, renal artery stenosis activates the renin–angiotensin–aldosterone system (RAAS), causing refractory hypertension. Yet, DES revascularization in renal arteries also shows elevated restenosis rates, particularly in smaller vessels, which can reactivate RAAS and impair systemic function [13].

Although the challenges are distinct, the underlying device deficiencies are common: unresolved modulation of endothelial repair and poor mechanical matching with dynamic anatomies. This is a multifactorial design challenge where AI-powered generative algorithms can excel. Instead of relying on iterative engineering, these tools can explore vast parameter spaces to optimize device geometries for organ-specific biomechanics. For example, one could create a patient-specific “digital twin” of a renal artery from computed tomography angiography data, then virtually test hundreds of AI-generated stent designs under simulated cardiac cycles. This in silico trial could not only predict immediate mechanical stress (ΨSTS) but also forecast long-term flow disturbance and its impact on endothelial health (ΦLTC) before a physical device is ever made. Extant metallic stents, due to their rigidity, predispose to both short-term stress concentration and long-term restenosis—a clear failure to achieve high suitcordance.

Analogously, long-dwelling inferior vena cava filters risk fracture and thrombosis; aspiration catheters for pulmonary embolism can cause vascular injury [14–16]. These examples all showcase a disruption of one or more of the vascular ecological equilibria. AI-driven risk models could prospectively identify patients at high risk for these complications, enabling more personalized device selection. The “low suitcordance dilemma” is thus a ubiquitous challenge for most deployed panvascular interventional devices.

Clinically, our imperative must extend beyond organ-level repair to encompass systemic health. We must broaden the panvascular framework to include the liver, kidneys, lungs, and spleen, and prioritize high-suitcordance enhancements in their corresponding interventional devices.

## Xinsorb and Polymer-TAVR: Clinical Blueprints for High-Suitcordance Device Design

In practice, our team’s development of the Xinsorb bioresorbable scaffold provides a compelling paradigm for creating high-suitcordance devices. It is important to acknowledge that the field of bioresorbable vascular scaffolds (BVS) has faced challenges: first-generation devices like Absorb BVS showed higher rates of late scaffold thrombosis, temporarily dampening enthusiasm [17]. However, through meticulous optimization, Xinsorb represents a new generation that addresses these shortcomings. It was engineered using finite element analysis and machine learning models to simulate stress distribution across the scaffold structure during all procedural stages—from crimping into the delivery catheter to expansion within the vessel—and to assess its fatigue resistance under postimplant pulsatile loading. This computational optimization of its mechanical architecture ensures robust short-term suitability (ΨSTS) by supporting the vessel. At the same time, it employs a fully degradable polylactic acid matrix, whose progressive degradation averts the long-term risks of a permanent metallic “cage”, facilitating physiological restoration and ensuring high long-term concordance (ΦLTC). Five-year clinical follow-up data validate its safety and efficacy, demonstrating near-complete strut absorption and sustained luminal patency, thereby embodying the attributes of high suitcordance [18].

Concurrently, the world’s first polymer-valve TAVR procedure, led by Academician Ge Junbo’s team, exemplifies another high-suitcordance innovation. Compared to traditional animal pericardial leaflets prone to calcification, by precisely tailoring the polymer’s mechanical properties and biocompatibility, the device not only achieves immediate hemodynamic improvement postimplantation (ΨSTS) but also ensures long-term biological integration with host tissue (ΦLTC). This extends its designed durability by 2- to 3-fold over traditional products, thereby remarkably reducing the patient’s risk of reoperation. This achievement powerfully demonstrates the practical value of the high-suitcordance framework and the 3 ecological equilibria theory in the interventional treatment of complex structural heart disease [19].

These advancements adhere to high-suitcordance principles. They provide a translatable framework for other organs. For instance, renal artery stents must balance radial strength with compliance. The adoption of fully bioresorbable materials is an emerging consensus. Drawing from the Xinsorb and polymer-valve TAVR paradigms, the path forward involves tailoring polymer coatings and drug loads to achieve controllable degradation and release kinetics, thereby realizing the goal of “high suitcordance”.

## Toward a New Era of Systemic Intervention: The Path to High Suitcordance via Interdisciplinary Integration

The future of panvascular medicine will pivot on high-suitcordance devices, driven by an interdisciplinary fusion of materials science, biomechanics, and AI. Specifically, AI will accelerate the discovery of novel biomaterials through virtual screening, enable generative design of patient-specific devices, and create “digital twin” simulations to predict suitcordance before implantation [20].

Imagine a scenario in which a coronary heart disease patient requires a personalized stent: AI first performs virtual screening based on the patient’s biomarker data, such as genome and proteome, to identify novel polymer materials with optimal biocompatibility and predictable degradation rates. Subsequently, leveraging generative design and utilizing precise 3-dimensional vascular geometry, plaque distribution, and mechanical properties obtained from coronary computed tomography angiography (CTA), it automatically creates a perfectly fitting stent mesh structure. In this process, digital twin technology serves as the core “proactive testing ground”—it integrates the patient’s CTA images and real-time hemodynamic data to construct a high-fidelity virtual replica of the vascular system. Within this digital twin, using physics engines and computational fluid dynamics to simulate the mechanical expansion of the stent implantation and blood flow dynamics, machine learning algorithms can predict device–tissue interactions, assess postoperative thrombosis risks, and evaluate long-term repair outcomes in advance. This allows for precise preoperative prediction and optimization of the solution’s suitability, mitigating potential risks in the virtual realm, and ultimately achieving a high degree of suitcordance between the interventional device and the individual patient across biological, geometrical, and functional dimensions. This will transition interventional therapy from isolated repair to synergistic management, rebalancing the postintervention vascular ecology [3].

As medicine shifts from treating isolated diseases to managing systemic health over a lifetime, we must transcend single organ dilemmas. Guided by AI-integrated platforms, we must expand the panvascular concept to include all full-watershed organs and relentlessly pursue higher suitcordance in device design. Only then can we safeguard both short-term suitability and long-term concordance, comprehensively restoring the body’s mechanical, cellular, and physicochemical equilibria and forging new paths in humanity’s confrontation with panvascular diseases.

## References

[1] Zhou X, Yu L, Zhao Y, Ge J. Panvascular medicine: An emerging discipline focusing on atherosclerotic diseases. Eur Heart J. 2022;43(43):4528–4531. 35947920 10.1093/eurheartj/ehac448

[2] Fabris E, Korjian S, Coller BS, Ten Berg JM, Granger CB, Gibson CM, van ’t Hof AWJ. Pre-hospital antiplatelet therapy for STEMI patients undergoing primary percutaneous coronary intervention: What we know and what lies ahead. Thromb Haemost. 2021;121(12):1562–1573. 33677829 10.1055/a-1414-5009 PMC8604087

[3] You L, Shen L, Ge J. The foundation and development of China’s National Basic Science Center for panvascular interventional complex systems: Pioneering device-vessel suitcordance research. Eur Heart J. 2025;46(35):3400–3403. 40464743 10.1093/eurheartj/ehaf418

[4] You L, Luo Y, Cheng Q, Shen L, Ge J. High-suitcordance intelligent fibers for panvascular disease monitoring-intervention. Adv Fiber Mater. 2025;7(4):1042–1072.

[5] Tapeinos C, Gao H, Bauleth-Ramos T, Santos HA. Progress in stimuli-responsive biomaterials for treating cardiovascular and cerebrovascular diseases. Small. 2022;18(36): Article e2200291. 35306751 10.1002/smll.202200291

[6] Soto-Gutierrez A, Gough A, Vernetti LA, Taylor DL, Monga SP. Pre-clinical and clinical investigations of metabolic zonation in liver diseases: The potential of microphysiology systems. Exp Biol Med. 2017;242(16):1605–1616. 10.1177/1535370217707731 PMC5661767 28467181

[7] Li Y, Liu Y, Liu S, Gao M, Wang W, Chen K, Huang L, Liu Y. Diabetic vascular diseases: Molecular mechanisms and therapeutic strategies. Signal Transduct Target Ther. 2023;8(1):152. 37037849 10.1038/s41392-023-01400-z PMC10086073

[8] Iwakiri Y. Endothelial dysfunction in the regulation of cirrhosis and portal hypertension. Liver Int. 2012;32(2):199–213. 21745318 10.1111/j.1478-3231.2011.02579.x PMC3676636

[9] Zoccali C, Vanholder R, Massy ZA, Ortiz A, Sarafidis P, Dekker FW, Fliser D, Fouque D, Heine GH, Jager KJ, et al. The systemic nature of CKD. Nat Rev Nephrol. 2017;13(6):344–358. 28435157 10.1038/nrneph.2017.52

[10] Jourde-Chiche N, Fakhouri F, Dou L, Bellien J, Burtey S, Frimat M, Jarrot PA, Kaplanski G, Le Quintrec M, Pernin V, et al. Endothelium structure and function in kidney health and disease. Nat Rev Nephrol. 2019;15(2):87–108. 30607032 10.1038/s41581-018-0098-z

[11] Ravarotto V, Bertoldi G, Stefanelli LF, Nalesso F, Calò LA. Pathomechanism of oxidative stress in cardiovascularrenal remodeling and therapeutic strategies. Kidney Res Clin Pract. 2022;41(5):533–544. 36239057 10.23876/j.krcp.22.069 PMC9576462

[12] Naidu S, Alzubaidi S, Knuttinen G, Patel I, Fleck A, Sweeney J, Aqel B, Larsen B, Buras M, Golafshar M, et al. Treatment of hepatic artery stenosis in liver transplant patients using drug-eluting versus bare-metal stents. J Clin Med. 2021;10(3):380. 33498286 10.3390/jcm10030380 PMC7863956

[13] Jundt MC, Takahashi EA, Harmsen WS, Misra S. Restenosis rates after drug-eluting stent treatment for stenotic small-diameter renal arteries. Cardiovasc Intervent Radiol. 2019;42(9):1293–1301. 31267151 10.1007/s00270-019-02264-z PMC6679807

[14] Ferro EG, Mackel JB, Kramer RD, Torguson R, Whatley EM, O’Connell G, Pullin B, Watson NW, Li S, Song Y, et al. Postmarketing surveillance of inferior vena cava filters among US Medicare beneficiaries: The SAFE-IVC study. JAMA. 2024;332(24):2091–2100. 39504004 10.1001/jama.2024.19553 PMC11541742

[15] Giri J, Sista AK, Weinberg I, Kearon C, Kumbhani DJ, Desai ND, Piazza G, Gladwin MT, Chatterjee S, Kobayashi T, et al. Interventional therapies for acute pulmonary embolism: Current status and principles for the development of novel evidence: A scientific statement from the American Heart Association. Circulation. 2019;140(20):e774–e801. 31585051 10.1161/CIR.0000000000000707

[16] Götzinger F, Lauder L, Sharp ASP, Lang IM, Rosenkranz S, Konstantinides S, Edelman ER, Böhm M, Jaber W, Mahfoud F. Interventional therapies for pulmonary embolism. Nat Rev Cardiol. 2023;20(10):670–684. 37173409 10.1038/s41569-023-00876-0 PMC10180624

[17] Stone GW, Kereiakes DJ, Gori T, Metzger DC, Stein B, Erickson M, Torzewski J, Kabour A, Piegari G, Cavendish J, et al. 5-year outcomes after bioresorbable coronary scaffolds implanted with improved technique. J Am Coll Cardiol. 2023;82(3):183–195. 37207924 10.1016/j.jacc.2023.05.003

[18] Wu Y, Shen L, Yin J, Chen J, Ge L, Ge J. 5 years of serial intravascular imaging outcomes of XINSORB sirolimus-eluting bioresorbable vascular scaffold. JACC Cardiovasc Interv. 2019;12(6):602–603. 30660461 10.1016/j.jcin.2018.11.029

[19] Ge J, Zhou D, Zhang X, Hou S, Chen S, Jin Q, Pan W, Li W, Pan C, Qian J. Preliminary implantation of a novel TAVR device with polymeric leaflets for symptomatic calcific aortic disease. JACC Case reports. 2023;17:101901. 37496722 10.1016/j.jaccas.2023.101901 PMC10366542

[20] Hang R, Yao X, Bai L, Hang R. Evolving biomaterials design from trial and error to intelligent innovation. Acta Biomater. 2025;197:29–47. 40081552 10.1016/j.actbio.2025.03.013

